# Exploring the effects of the dietary fiber compound mediated by a longevity dietary pattern on antioxidation, characteristic bacterial genera, and metabolites based on fecal metabolomics

**DOI:** 10.1186/s12986-024-00787-y

**Published:** 2024-04-04

**Authors:** Fengcui Shi, Qingli Liu, Dayong Yue, Yanan Zhang, Xueying Wei, Ying Wang, WenJian Ma

**Affiliations:** 1 School of Chemical and Biological Engineering, Qilu Institute of Technology, Shandong, China; 2 College of Biotechnology, Tianjin University of Science and Technology, Tianjin, China

**Keywords:** Longevity dietary, Dietary fiber compound, Aging, Characteristic bacterial genera, Metabolites

## Abstract

**Background:**

Age-related dysbiosis of the microbiota has been linked to various negative health outcomes. This study aims to investigate the effects of a newly discovered dietary fiber compound (DFC) on aging, intestinal microbiota, and related metabolic processes. The DFC was identified through in vitro fermentation screening experiments, and its dosage and composition were determined based on a longevity dietary pattern.

**Methods:**

Aged SPF C57BL/6 J mice (65 weeks old) and young mice (8 weeks old) were divided into three groups: a subgroup without dietary fiber (NDF), a low DFC dose subgroup (LDF, 10% DFC), and a high DFC dose subgroup (HDF, 20% DFC). The total antioxidant capacity (T-AOC), total superoxide dismutase (T-SOD) activity, malondialdehyde (MDA) content, and glutathione peroxidase (GSH-Px) activity in liver and serum samples of the mice were measured according to the manufacturer’s protocol. The expression levels of characteristic bacterial genera and fecal metabolite concentrations in mice were determined using quantitative real-time PCR (qPCR) and nuclear magnetic resonance hydrogen spectroscopy (^1^H NMR). Metabolomics analysis was further conducted to identify biological functions and potential pathways related to aging.

**Results:**

After an 8-weeks dietary intervention, DFC supplementation significantly attenuated age-related weight loss, organ degeneration, and oxidative stress. And promoted the growth of *Lactobacillus* and *Bifidobacterium* and inhibited the growth of *Escherichia coli* (*E. coli*) and *Bacteroides* (*p* < 0.05) in the intestinal tracts of aged mice. Metabolomic analysis identified glycolipid and amino acid metabolic pathway biomarkers associated with aging that were differentially regulated by DFC consumption. Correlation analysis between the identified microbial flora and the biomarkers revealed potential mechanistic links between altered microbial composition and metabolic activity with aging markers.

**Conclusions:**

In conclusion, this study revealed an important mechanism by which DFC consumption impacts healthspan and longevity, shedding light on optimizing dietary fiber or developing fiber-based interventions to improve human health.

**Supplementary Information:**

The online version contains supplementary material available at 10.1186/s12986-024-00787-y.

## Introduction

Bama County in Guangxi is a world-renowned longevity town, where the phenomenon of longevity is highlighted [1]. Our research team conducted a lot of research on the dietary characteristics of the longevity population in the early stage, and found that the traditional diet in the Bama area is mainly based on the food intake of plant foods, such as coarse grains (Taro, hemp, corn, cassava, etc.), vegetables (Pumpkin seedlings, sweet potato leaves, water spinach, amaranth, wild rattan, etc.), and fruits (Kumquat, plantain, etc.) and the intake of dietary fiber is relatively high [2, 3], and dietary fiber is closely linked to the formation of longevity phenomenon, and longevity is closely related to the structure of the intestinal microenvironment or intestinal flora [4]. A specific bacterial community pattern and signature taxa of long-lived people were found in long-lived families, such as the enrichment of *Enterobacteriaceae* in all age groups and the higher abundances of *Christensenellaceae*, *Verrucomicrobiaceae*, *Porphyromonadaceae*, *Rikenellaceae*, *Mogibacteriaceae*, and *Odoribacteraceae* in long-lived elderly [4]. Therefore, exploring the relationship between dietary fiber and aging, intestinal microenvironment, or intestinal flora is of great significance to longevity research. Our team has summarized the dietary pattern reflecting the dietary characteristics of the longevity population and found that this pattern has good anti-aging effects and development potential [5, 6], but the extent to which dietary fiber plays a role in it has not been studied.

Age-related dysbiosis of the gut microbiota, characterized by reduced diversity and altered composition, has been linked to age-related metabolic dysregulation and increased risk for various chronic diseases [7]. Dietary fiber intake is known to benefit gut microbiota and metabolic homeostasis, but the impacts of specific fiber types and combinations on microbiota-related biomarkers of aging are not fully understood [8]. Elucidating the mechanisms by which dietary fiber compounds modulate the gut microbiome and related metabolic changes is of great interest for advancing longevity research. Previous studies have examined the effects of individual dietary fibers or simple combinations on health outcomes. Dietary fiber intake has been shown to lower blood sugar and cholesterol [9, 10], relieve constipation [11], and improve gut barrier function and immunity [12]. For example, ginseng soluble dietary fiber can regulate the structure of the intestinal flora and improve glucose and lipid metabolism in rats [13]. Walnut meal fiber increased the production of short-chain fatty acids and gut microbiota diversity [14]. A mixture of galacto-oligosaccharides, inulin, polydextrose, and wheat bran balanced bacterial growth by modulating the expression of diamine oxidase (DAO) and trimethylamine nitrogen oxides (TMAO) [15]. However, most of these reports focused on one or a few combinations of dietary fibers. Studies on more complex dietary fiber compounds from a specific traditional dietary pattern high in diverse fiber sources have been rare.

In this study, three DFCs were constructed based on the longevity dietary pattern in the preliminary stage and screened for effects by in vitro fermentation experiments, and it was found that compounding fruits, vegetables, and grains exerted a greater regulatory effect on intestinal health [16], and it is not yet known how much of a role this DFC plays in vivo. The current study investigated the effects of DFC on aging phenotypes, characteristic bacterial genera, and metabolite changes in mice, to better provide theoretical support for improving human health through supplementing dietary fiber.

## Materials and methods

### Preparation of dietary fiber compound (DFC)

DFC was prepared according to previously published recipes [2, 3]. The primary DFC mixture contained sweet potato leaf, wolfberry leaf, bitter oatmeal, cabbage, banana, plantain, mandarin orange, and taro at a weight ratio of 1.4:1.3:0.98∶0.8:11.5:5.4:1.3:7.4. After sufficient mixing, distilled water was added according to 15 mL/g liquid-to-material ratio, 0.3% α-amylase (CAS9000-90-2, 4000 U/g) was added, the pH was adjusted to 5.0, 240W ultrasonic power was set to assist the enzymatic hydrolysis for 20 min, and the extract was placed in water bath and extracted under the condition of 90 min, 80 °C, and then the enzyme was inactivated in the water bath of 100 °C for 10 min. After cooling to room temperature, centrifuge the mixture at 8000 rpm for 15 min, and the precipitate was freeze-dried under vacuum to obtain the insoluble dietary fiber (IDF) fraction. The supernatant was precipitated with 4 times the volume of 95% ethanol, centrifuged at 8000 rpm for 10 min, and the precipitate was freeze-dried under vacuum to obtain the soluble dietary fiber (SDF) fraction. Finally, the extracted IDF and SDF were directly mixed according to the actual extraction ratio to obtain the final DFC mixture. Because of the number of abbreviations used in this study, we summarized the abbreviations in the Additional file 1: Table S1.

### Animal grouping and sample collection

The experimental protocol and experiments were approved by the Ethics Committee of Guangxi University (Production License No: gxdxdwll01). Seventy-two SPF C57BL/6 J grade mice were purchased from Beijing Specific Bio-technology Co., Ltd (Test Animal Production License No.: SCXK (Beijing) 2019-0010). The basic feed used in this study is XTADMOO1 purified feed in the maintenance period. It can be used as the basic feed. Based on its formula, various nutrients are added and reduced. The different physiological states of experimental rats under different nutrient concentrations are explored and used as the control group (NDF). Thirty-six 65-week-old mice were constructed as the aged mice model (aged group, AG) and randomly divided into 3 subgroups (n = 12 each, half of males and females): no dietary fiber (AG-NDF), low DFC dose (10% DFC, AG-LDF), and high DFC dose (20% DFC, AG-HDF). Thirty-six 8-week-old mice served as young controls (young group, YG) and were also divided into 3 subgroups (n = 12 each, half of males and females): no dietary fiber (YG-NDF), low DFC dose (10% DFC, YG-LDF) and high DFC dose group (20% DFC, YG-HDF). Details of the feed formulation for each group of mice were summarized in Additional file 2: Table S1. Mice (female and male) were raised separately, with 6 in each cage. They were raised under light/dark cycling conditions of 24 ± 2 °C, relative humidity of 55 ± 5%, and 12 h, and were able to freely obtain food and water. Before the dietary intervention, all mice received a libitum diet and water for 1 week. After this acclimatization, the 8-week intervention was initiated. At the intervention end, mice were fasted and dehydrated for 12 h. Following anesthesia, heart, liver, spleen, lungs, kidneys, cecum contents, and colon contents were collected and stored in a − 80 °C.

### Measurement of body weight and food intake of mice

Fecal samples were collected at week 1 and week 8 by massaging the abdomen. Body weight and food intake were recorded weekly. Fecal samples were stored in a refrigerator at − 80 °C until analysis. Before weighing, mice were fasted for 12 h.

### Determination of organ coefficient in mice

The weight of each part of the organs of the mice was weighed and calculated according to the following formula:

1 $${\text{Organ}}\,\,{\text{coefficient/\% }} = \frac{{{\text{m}}_{1} }}{{{\text{m}}_{2} }} \times 100$$

m_1_ is the weight of the organ and m_2_ is the final body weight of the mouse.

### Determination of mouse liver and serum antioxidant capacity

Total antioxidant (T-AOC) capacity, total superoxide dismutase (T-SOD) activity, malondialdehyde (MDA) content, and glutathione peroxidase (GSH-Px) activity were determined in mouse liver and serum samples according to the manufacturer's protocols (Nanjing Jiancheng Institute of Bioengineered Enzymes, Nanjing, China).

### Extraction of DNA from mouse feces

Bacterial DNA was extracted from fecal samples using a fecal genomic DNA extraction kit (Solarbio, Beijing, China). Nucleic acids were obtained from 200 mg of fecal samples and eluted in 90 µL of elution buffer. DNA concentration was quantified using an infinite M200 pro microtiter detector (Tecan, Männedorf, Switzerland). Extracted DNA samples were stored at − 80 °C until further analysis.

### Determination of qPCR

Fecal DNA samples were quantified by qPCR using a Roche Light Cycler 96 qPCR instrument (Roche Diagnostics Co., Ltd., Basel, Switzerland) with 96-well optical plates. Primers used are listed in Table 1. Relative quantification was performed using the comparative Ct method, and the formula is as follows:

2 $$Relative\,expression\,level = 2^{{ - \left\{ {\left( {{\text{Ct}}\,\,{\text{value}}\,\,{\text{of}}\,\,{\text{target}}\,\,{\text{gene}}\,\,{\text{to}}\,\,{\text{be}}\,\,{\text{tested}} - {\text{Ct}}\,\,{\text{value}}\,\,{\text{of}}\,\,{\text{internal}}\,\,{\text{gene}}\,\,{\text{to}}\,\,{\text{be}}\,\,{\text{tested}}} \right) - \left( {{\text{Ct}}\,\,{\text{value}}\,\,{\text{of}}\,\,{\text{control}}\,\,{\text{target}}\,\,{\text{gene}} - {\text{Ct}}\,\,{\text{value}}\,\,{\text{of}}\,\,{\text{control}}\,\,{\text{internal}}\,\,{\text{reference}}\,\,{\text{gene}}} \right)} \right\}}}$$

**Table 1:** Primer sequences of five species

Bacteria	Primer Sequence (5′-3′)	References
Total intestinal flora	F:ACTCCTACGGGAGGCAGCAG R: ATTACCGCGGCTGCTGG	[17]
*E. coli*	F:GTTAATACCTTTGCTCATTGA R:ACCAGGGTATCTTAATCCTGTT	[18]
*Lactobacillus*	F:AGCAGTAGGGAATCTTCCA R:CACCGCTACACATGGAG	[19]
*Bifidobacterium*	F:TCGCGTC(C/T)GGTGTGAAAG R:CCACATCCAGC(A/G)TCCAC	[20]
*Bacteroides*	F:CTGAACCAGCCAAGTAGCG R:CCGCAAACTTTCACAACTGACTTA	[21]

### Determination of metabolites in mouse feces by ^1^H NMR

A homogenized 50 mg aliquot was placed into a 2.0 mL safe-lock tube (Eppendorf, Hamburg, Germany). Then, 500 μL PBS/D_2_O buffer (0.1 M, pH 7.4) was added, containing 10% D_2_O (v/v) and 0.005% TSP (w/v). The mixture was repeatedly frozen and thawed in liquid nitrogen three times, homogenized for 60 s, and then centrifuged (12,000 g, 10 min, 4 °C). Collect the supernatant and repeat the extraction process. The pooled supernatants were collected and centrifuged (12,000 g, 15 min, 4 °C) and 550 μL was transferred to a 5 mm NMR tube for detection.

^1^H NMR spectra were acquired on a Bruker Avance 500 MHz NMR spectrometer at 298 K using a water pre-saturated standard one-dimensional NOESYPR ^1^D pulse sequence (recycle delay − 90° − t_1 − _90° − t_m − _90°—acquisition) with water suppression. Acquisition parameters were relaxation delay 2.0 s, mixing time 0.1 s, 64 scans, 65,536 data points, spectral width 10,000 Hz. Free induction decays were multiplied by 0.5 Hz exponential line broadening before Fourier transformation. Spectra were referenced to TSP (δ = 0.0 ppm), phase and baseline corrected and integrated over the spectral regions of 0.00–9.00 ppm into 0.001 ppm bins. The water region (4.70–5.10 ppm) was excluded data normalized before analysis.

### Metabolomics analysis

Multivariate analysis of NMR data was performed using the metaboanalyst website (https://www.metaboanalyst.ca/). Unsupervised principal component analysis (PCA) was used to examine any intrinsic clustering. Orthogonal partial least squares discriminant analysis (OPLS-DA) was done in SIMCA 14.1 (Umetrics, Sweden) software to maximize the separation of fecal metabolites between groups after DFC intervention. OPLS-DA models were validated by 200 conditions using the permutation tests. Metabolic pathway enrichment and pathway analysis using the kyoto encyclopedia of genes and genomes (KEGG) database identified altered pathways based on differential metabolites. Correlation heatmaps and network analysis were generated in the omicstudio website (https://www.omicstudio.cn/tool) to determine associations between the characterized microbiota and metabolites.

### Statistical analysis

Results were expressed as mean ± standard deviation. All statistical analyses were performed using SPSS 22.0 software (SPSS Inc., Chicago, IL). One-way analysis of variance (ANOVA) was used for multiple comparisons. Differences were considered statistically significant when *p* < 0.05.

## Results

### DFC prevents age-related weight loss and enhances food utilization

Age-related loss of muscle mass contributes significantly to frailty and mortality risk. We therefore firstly tested whether DFC could attenuate weight loss in aged mice with 8 weeks of dietary intervention. There was no significant change in body weight for either YG or AG groups in the first week following DFC intervention (*p* > 0.05) (Table 2). However, by week 8, the AG-NDF group showed decreased body weight compared to baseline (− 1.60 ± 1.56 g, *p* < 0.05), indicating age-related weight loss. In contrast, DFC supplementation attenuated this weight loss in a dose-dependent manner, with both AG-LDF (+ 3.10 ± 2.45 g, *p* < 0.05) and AG-HDF (+ 5.30 ± 2.04 g, *p* < 0.05) groups showing significantly increased body weight compared to AG-NDF controls. The body weight gains in AG-HDF mice were also significantly higher compared to AG-LDF (*p* < 0.05).

**Table 2:** Changes in body weight of mice in each group after 8 weeks of intervention

Intervention Time/Week	Group					
	YG-NDF	YG-LDF	YG-HDF	AG-NDF	AG-LDF	AG-HDF
1	26.5 ± 2.37^bB^	26.7 ± 2.26^bB^	25.6 ± 2.36^bD^	35.8 ± 3.05^aC^	35.2 ± 2.22^aD^	36.6 ± 3.41^aD^
2	26.9 ± 2.16^cB^	26.0 ± 3.28^cB^	25.1 ± 2.29^dD^	36.2 ± 2.46^bC^	35.6 ± 3.46^bBD^	37.2 ± 2.62^aD^
3	26.6 ± 3.33^cB^	26.5 ± 2.37^cB^	24.9 ± 3.32^dAD^	35.8 ± 2.41^bBC^	36.8 ± 3.26^aAB^	37.7 ± 2.18^aCD^
4	27.6 ± 2.25^cA^	25.9 ± 2.10^dAB^	24.0 ± 2.63^dAC^	35.5 ± 2.51^bBC^	36.2 ± 2.21^aB^	37.5 ± 2.37^aC^
5	27.1 ± 3.09^dA^	25.2 ± 2.18^eAB^	24.8 ± 3.72^eAC^	35.2 ± 2.71^cBC^	36.8 ± 2.66^bAB^	38.5 ± 2.44^aB^
6	27.7 ± 2.27^cA^	25.1 ± 2.39^dAB^	23.3 ± 2.16^eB^	34.7 ± 2.49^bAB^	36.7 ± 2.40^aAB^	38.9 ± 2.38^aB^
7	27.4 ± 1.51^dA^	25.8 ± 2.55^dAB^	24.4 ± 2.25^eA^	34.6 ± 3.36^cAB^	36.8 ± 2.33^bAB^	39.2 ± 2.65^aAB^
8	27.6 ± 2.47^dA^	25.4 ± 3.47^dA^	24.0 ± 2.72^eAB^	34.2 ± 2.38^cA^	37.3 ± 3.36^bA^	39.5 ± 3.37^aA^

In the same column, different capital letters indicate that the same group has significant differences at different intervention times, *p* < 0.05. In the same line, different lowercase letters indicate significant differences between groups, *p* < 0.05

*YG* young control group, *AG* aged group, *NDF* no dietary fiber subgroup, *LDF* low *DFC* dose subgroup, *HDF* high DFC dose subgroup

**Fig. 1:**
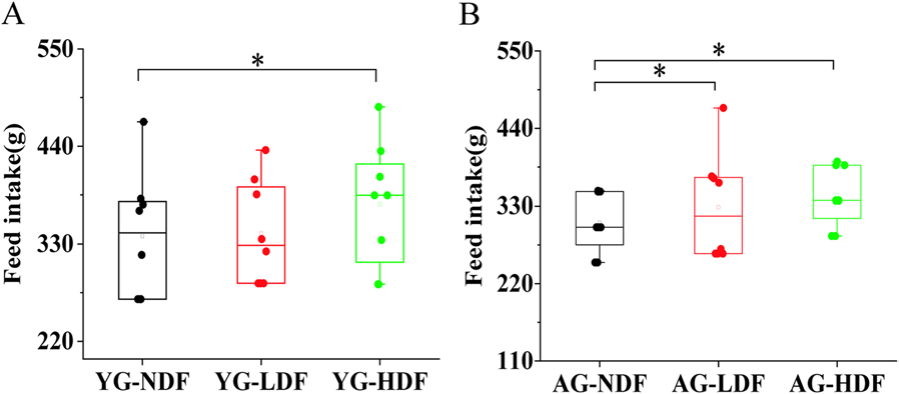
The changes in food intake of mice in each group after 8 weeks of intervention. **A** Food intake in each YG subgroup of mice. **B** Food intake in each AG subgroup of mice. *Note* *P < 0.05 **P < 0.01 vs NDF group

In young control mice (YG groups), DFC decreased body weight gain relative to YG-NDF controls, with enhancement more pronounced in YG-HDF (− 2.20 ± 1.84 g) versus YG-LDF (− 3.60 ± 2.38 g) groups (*p* < 0.05). Food intake followed a similar improvement with DFC in both aged and young groups (Fig. 1A, B), indicating food utilization efficiency was increased with DFC supplementation, particularly at higher doses. Overall, these data demonstrate that DFC attenuates age-associated weight loss and improves food utilization in mice.

### The impacts of DFC on organ coefficients in aged versus young mice

To assess changes in relative organ mass, organ coefficients (the ratio of organ weight to overall body weight) were determined.. The values of organ coefficients of mice after 8 weeks of DFC intervention were calculated and are shown in Table 3. Compared to YG-NDF controls, the organ coefficients of all examined organs were significantly decreased (*p* < 0.05) in AG-NDF mice. In the YG group, compared with the YG-NDF controls, the liver coefficient decreased significantly in the low-dose and high-dose DFC groups (p < 0.05), but there were no significant changes in other organ coefficients (*p* > 0.05). In the AG groups, heart, liver, spleen, and lung coefficients exhibited significant increases (*p* < 0.05) with both low and high-dose DFC versus AG-NDF controls. Kidney coefficients did not significantly differ between any AG subgroups (*p* > 0.05). These results demonstrate that organ decline occurred in aged mice to a certain extent, and DFC supplementation could increase the organ coefficient of aged mice, and the organ-protective effect of DFC appear somewhat more pronounced in higher doses of AG mice.

**Table 3:** Effect of DFC intervention on organ coefficients of mice in each group

Group	Organ coefficient				
	Cardiovascular	liver	Spleen	Lung	Kidney
YG-NDF	0.50 ± 0.06^b^	4.31 ± 0.84^b^	0.53 ± 0.22^a^	0.73 ± 0.12^b^	1.29 ± 0.11^a^
YG-LDF	0.51 ± 0.06^b^	3.59 ± 0.82^c^	0.60 ± 0.52^a^	0.73 ± 0.13^b^	1.34 ± 0.21^a^
YG-HDF	0.52 ± 0.07^b^	3.07 ± 1.78^c^	0.74 ± 1.34^a^	0.99 ± 0.20^a^	1.32 ± 0.47^a^
AG-NDF	0.43 ± 0.04^c^	3.28 ± 0.69^c^	0.21 ± 0.15^c^	0.52 ± 0.08^c^	1.12 ± 0.12^b^
AG-LDF	0.54 ± 0.08^b^	4.04 ± 1.80^b^	0.29 ± 0.09^b^	0.68 ± 0.13^ab^	1.25 ± 0.18^ab^
AG-HDF	0.60 ± 0.07^a^	5.24 ± 0.92^a^	0.34 ± 0.15^b^	0.76 ± 0.16^a^	1.28 ± 0.13^ab^

Different letters (a, b, c) in the same column indicate significant differences, *p* < 0.05

### Effects of DFC on antioxidant capacity in aged versus young mouse

**Fig. 2:**
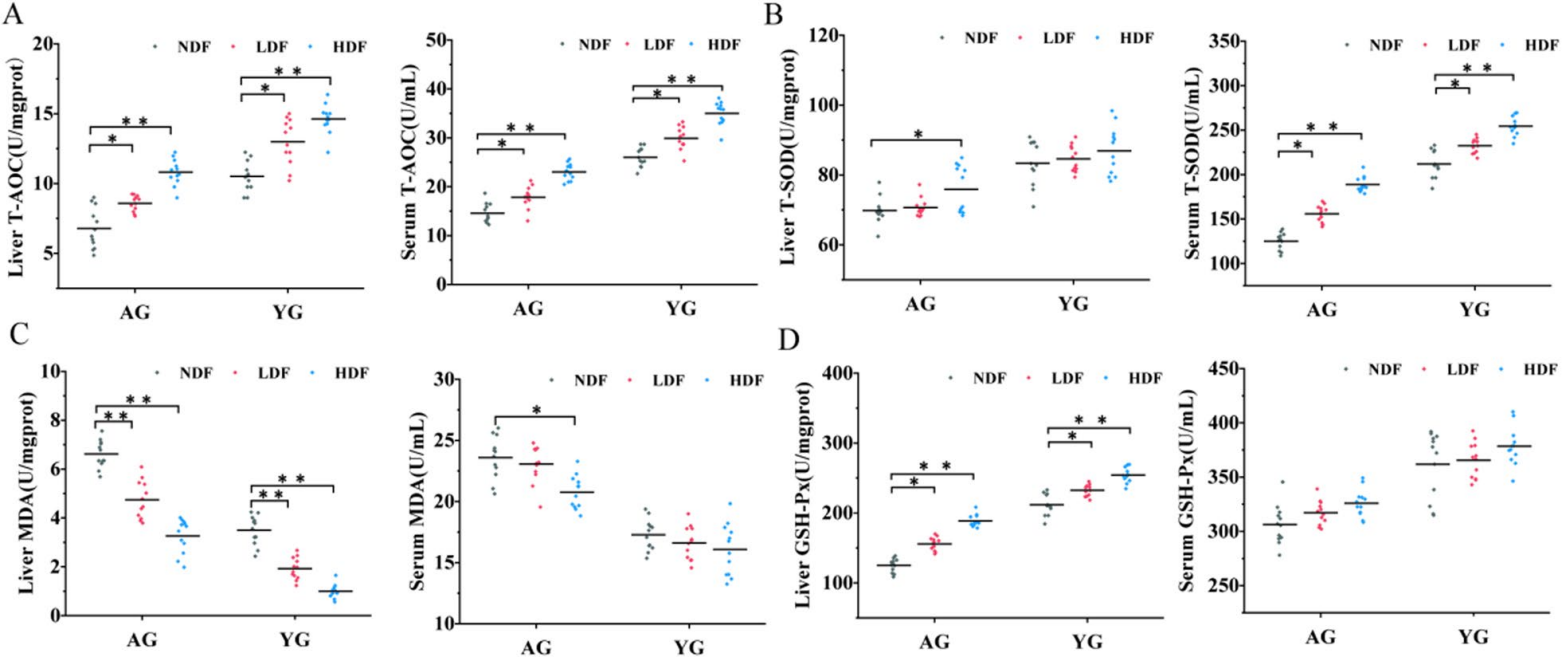
Effects of DFC intervention on antioxidant capacity in liver and serum of mice. Antioxidant capacity was assessed by measuring (**A**) T-AOC activity. (**B**) T-SOD activity. (**C**) MDA levels. (**D**) GSH-Px levels. *Note* *P < 0.05 **P < 0.01 versus NDF group

As oxidative stress plays a key role in the aging process, we hypothesized that DFC intervention might help suppress oxidative stress through increasing antioxidant capacity. Therefore, we examined the effects of DFC on antioxidant capacity in the livers and serum of young versus aged mice by measuring total antioxidant capacity (T-AOC), superoxide dismutase (T-SOD), and glutathione peroxidase (GSH-Px) that reflect antioxidant capacity, as well as malondialdehyde (MDA), a biomarker of oxidative stress (Fig. 2) [22, 23].

Compared to YG-NDF controls, T-AOC activities significantly increased in both the liver (76.51%) and serum (45.10%) of YG-HDF mice (*p* < 0.01). T-SOD activity also increased by 44.91% in YG-HDF serum (*p* < 0.01). MDA decreased by 77.90% in YG-HDF liver (*p* < 0.01). Liver GSH-Px increased by 63.26% in YG-HDF (*p* < 0.01). In AG mice, liver and serum T-AOC showed large significant increases of 95.64% and 115.05% in AG-HDF, respectively (*p* < 0.01). Liver T-SOD increased by 8.71% in AG-HDF (*p* < 0.05). Serum T-SOD increased by 77.55% in the AG-HDF (*p* < 0.01). MDA decreased by 70.76% in AG-HDF liver (*p* < 0.01) and 12.00% in serum (*p* < 0.05). Liver GSH-Px activity increased by 60.58% with AG-HDF (*p* < 0.01), but serum GSH-Px activity had no significant difference (*p* > 0.05). Collectively, DFC supplementation substantially improved antioxidant capacity in both young and aged mice, especially at high doses. The liver and serum of aged mice showed greater enhancements in T-AOC with DFC compared to young controls.

### DFC supplementation shifted Characteristic bacterial genera composition

**Fig. 3:**
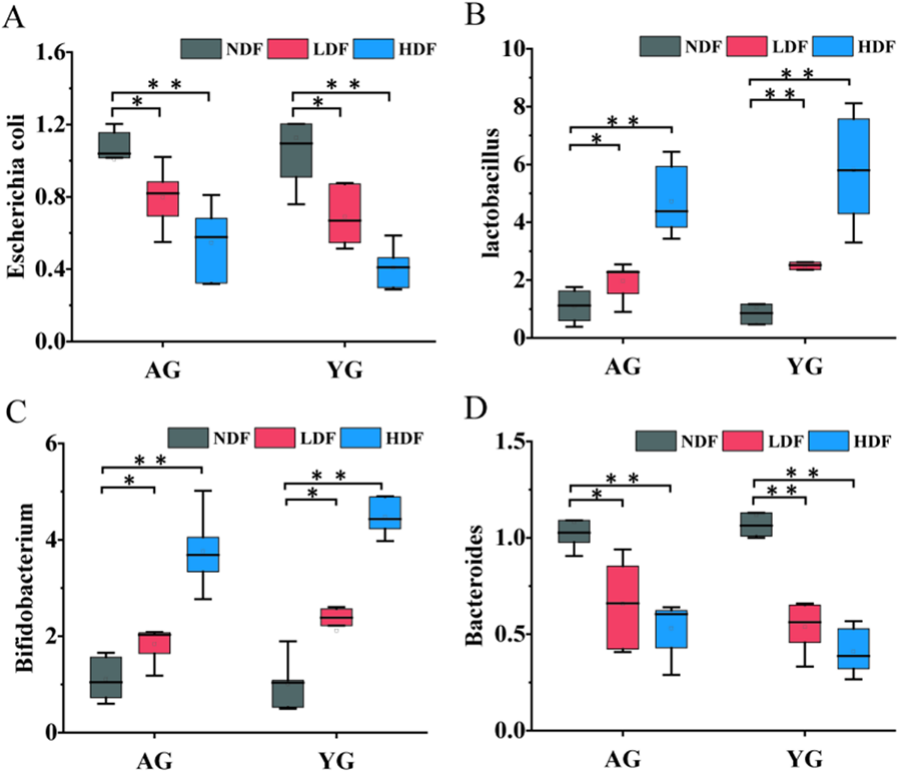
DFC supplementation alters characteristic bacterial genera composition in young and aged mice. Relative abundance of key gut bacterial genera was determined in the feces of young (YG) and aged (AG) mice after 8 weeks of DFC supplementation. (**A**) *E. coli*. (**B**) *Lactobacillus.* (**C**) *Bifidobacterium* (**D**) *Bacteroides.* Data shown as median and SD relative to respective NDF groups, analyzed by Student T-test. *Note* *P < 0.05 **P < 0.01 vs NDF group

As the gut microbiota plays an important role in health and can be impacted by diet and age, we evaluated the effects of 8 weeks of DFC supplementation on key gut bacterial groups in feces of YG and AG mice (Fig. 3). As we all know, when the amount of *E. coli* in the intestine is excessive, it will destroy the intestinal barrier and become an opportunistic pathogen [24]. Compared to NDF controls, the *E. coli* decreased with DFC treatment in both YG and AG groups. In AG mice, *E. coli* was significantly reduced by 20.84% in the LDF group (*p* < 0.05) and 45.62% in the HDF group (*p* < 0.01). In YG mice, it was significantly reduced by 38.66% (*p* < 0.05) in the LDF group and 63.64% (*p* < 0.01) in the HDF group. In the contrary, the beneficial genus *Lactobacillus* was significantly elevated by 128.46% (*p* < 0.01) in the YG-LDF group and 449.74% (*p* < 0.01) in the YG-HDF group. It was elevated in the AG groups as well by 79.31% (*p* < 0.05) in the AG-LDF group and 329.73% (*p* < 0.01) in the AG-HDF group. Another beneficial gut microbe *Bifidobacteria* was also elevated in both YG and AG groups upon DFC intervention, which was elevated by 109.05% (*p* < 0.05) in the YG-LDF group, 341.79% (*p* < 0.01) in the YG-HDF group, 66.34% (*p* < 0.05) in the AG-LDF group and 239.75% (*p* < 0.01) in the AG-HDF group. While being generally considered a beneficial genus of gut bacteria, however, the level of *Bacteroides* was significantly reduced by 39.22% (*p* < 0.01) in the YG-LDF group, 45.62% (*p* < 0.01) in the YG-HDF group, 52.00% (*p* < 0.05) in the AG-LDF group and 63.42% (*p* < 0.01) in the AG-HDF. It is known that some species of *Bacteroides* can become opportunistic pathogens if they spread beyond the gut and cause systemic infection [25]. Therefore, these results indicate that DFC supplementation, significantly increased beneficial bacteria and reduced potential pathogens in both young and aged mice, suggesting DFC may improve characteristic bacterial genera composition in aging.

### Identification of metabolite changes in mouse feces upon DFC intervention

**Fig. 4:**
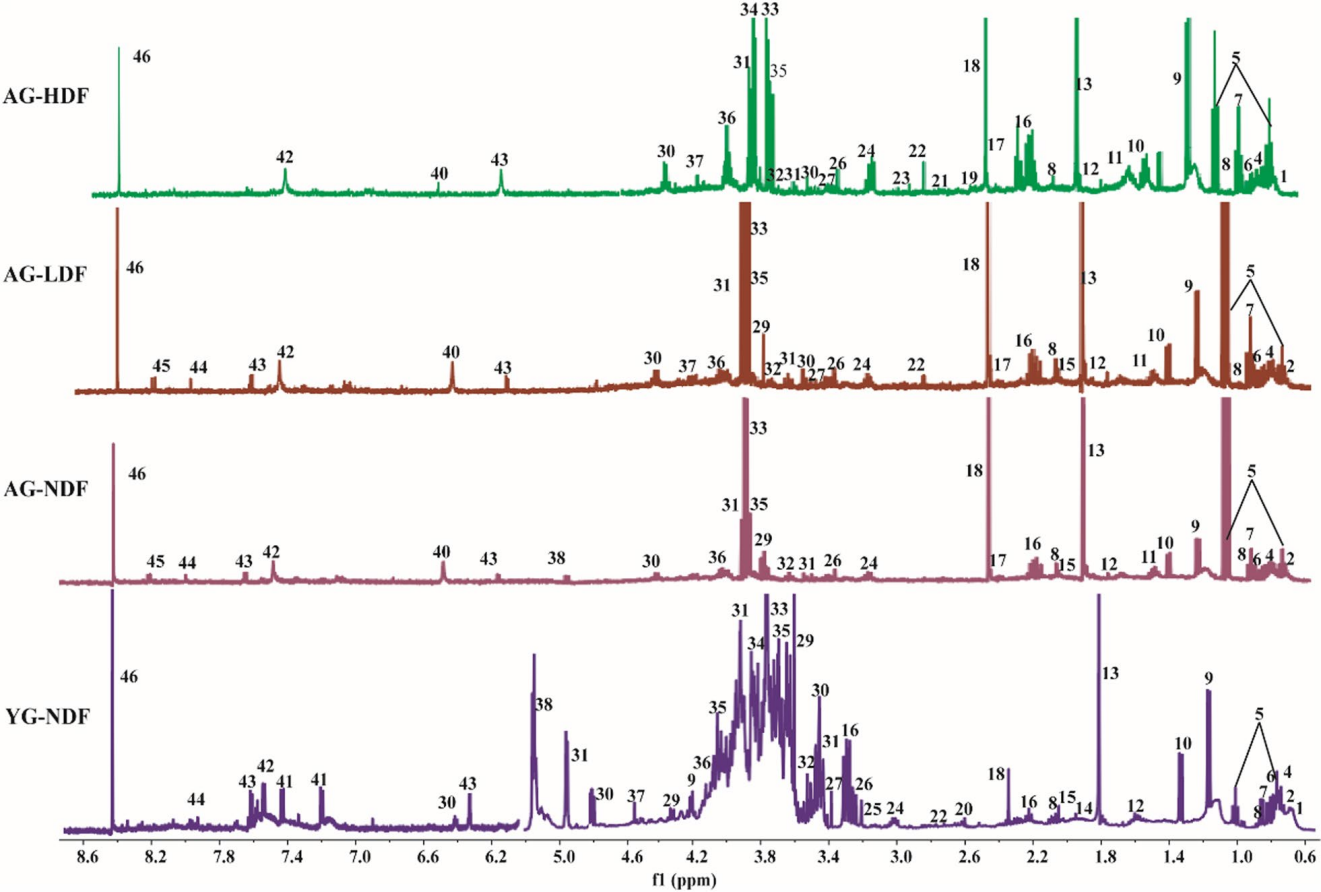
Representative ^1^H NMR metabolic spectrum of metabolites in mouse fecal samples

To investigate the metabolic alterations induced by DFC supplementation, we performed untargeted ^1^H NMR-based metabolomics analysis on fecal samples from mice after 8 weeks of DFC treatment. A representative metabolite ^1^H NMR spectrum in feces was shown in Fig. 4. A total of 46 metabolites were identified in mouse fecal samples, with chemical shifts mainly distributed in the range of 0.5–8.5 ppm, spanning diverse chemical classes including sugars, amino acids, fatty acids, organic acids, and others. Assignment of metabolites was confirmed using the human metabolome database (HMDB) (http://www.hmdb.ca/) and biological magnetic resonance bank (BMRB) (http://www.bmrb.wisc.edu/), which were calibrated and assigned in conjunction with references [26–28], and are summarized in Additional file 3: Table S1. The ^1^H signal in the 0.8–3.0 ppm region represents amino acids (leucine, valine, alanine, aspartic acid, proline, and lysine, etc.), short chain fatty acids (acetic acid, propionic acid, and butyric acid, etc.), organic carboxylic acids (bile acid, lactic acid, succinic acid, and citric acid, etc.), and other metabolites (dimethylamine, trimethylamine, and N-acetylglycoprotein, etc.). The ^1^H peak in the 3.0–5.5 ppm range mainly includes sugars (*α*-Glucose, *β*-Glucose, *α*-Galactose, and *β*-Galactose), amino acids (such as histidine, lysine, threonine, glycine, and serine), and other metabolites (such as choline and methanol). Finally, amino acids (tyrosine), organic acids (formic acid, fumarate), and other metabolites (inosine, uric acid, uracil, xanthine, and hypoxanthine, etc.) were found in the region of around 5.5–8.5 ppm.

**Fig. 5:**
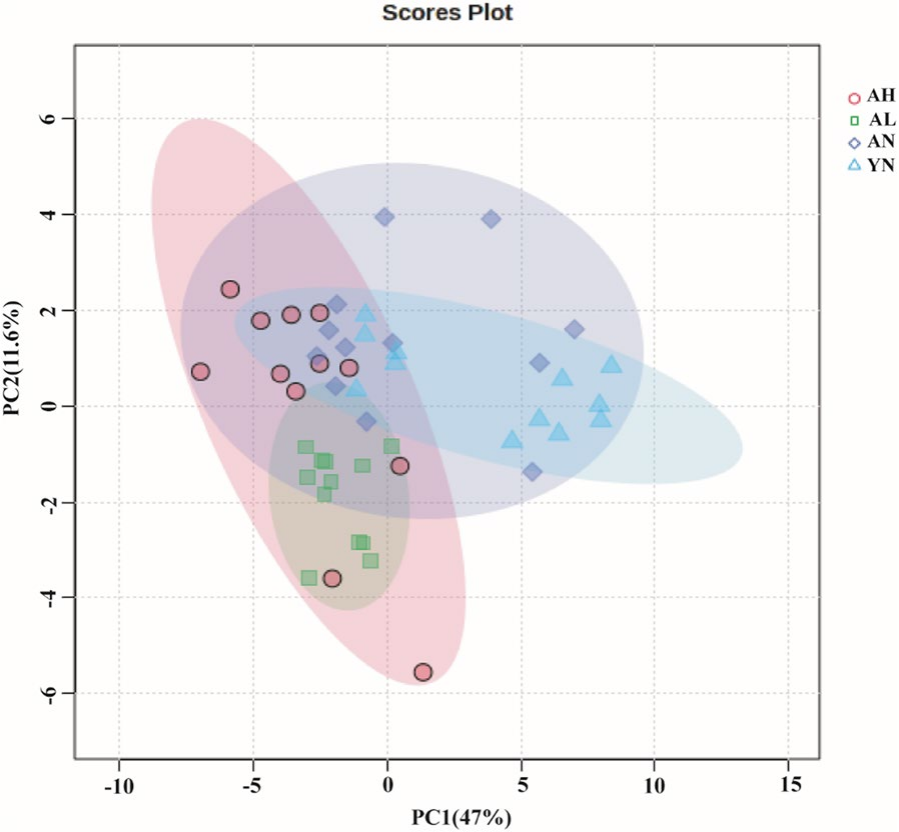
Scatter plot of PCA scores of fecal metabolites in mice after DFC intervention. PCA reveals dose-dependent and age-dependent changes in the fecal metabolomic profiles of mice in response to 8 weeks of dietary DFC intervention. *Note* AN = aging mice group on a no dietary fiber feed (aging control); AL = aging mice group on a low-dose DFC feed; AH = aging mice group on a high-dose DFC feed; YN = young mice group on a no dietary fiber feed group (negative control)

To characterize the overall metabolic profile in the feces of each mouse group, PCA was performed on the ^1^H NMR spectral data. As shown in Fig. 5, The PCA scores plot showed clustering of the samples according to the treatment group, with separation along the first principal component (PC1, 47%) distinguishing the young control, aging model, and DFC-treated aging groups. The second principal component (PC2, 11.6%) revealed the metabolic separation characteristics between the aging model group fed basal diet versus the low and high-dose DFC groups.

### Altered fecal metabolites in mice following DFC intervention

While PCA demonstrated clear metabolic changes between different groups, there was some overlap in the PCA scores clusters. To better discriminate the metabolic variations between groups, OPLS-DA was applied. As shown in Additional file 3: Fig. S1, the R^2^Y and Q^2^ values were greater than 0.9 for all pair-wise comparisons between groups: R^2^Y = 0.994 and Q^2^ = 0.991 between the AG and YG group; R^2^Y = 0.994 and Q^2^ = 0.987 between the LDF and NDF group; R^2^Y = 0.994 and Q^2^ = 0.987 between the HDF and NDF group. When these values are greater than 0.5, it is considered a high degree of fitting. The closer the values to 1, the more reliable the OPLS-DA model. Therefore, the above results indicate the OPLS-DA models have excellent predictive ability and can effectively explain the significant differences in fecal metabolites among different mouse groups.

A permutation test was conducted under 200 conditions for further interactive validation, which confirmed the robustness of the OPLS-DA models (as shown in Additional file 3: Fig. S1). Based on the log_2_Fold Change (log_2_FC) value combined with variable importance in projection (VIP) > 1 and *p* < 0.05, differential metabolites and up-regulation ranges were identified (Table 4). Between AG and YG, 9 metabolites were altered including increased uric acid, threonine, inosine, fumarate, leucine, pyruvate, and aspartic acid, and decreased propionic acid and butyric acid in AG. Between LDF and NDF, 12 metabolites changed significantly with propionic acid, butyrate, proline, glutamate, serine, fumarate, and citric acid were elevated in LDF while uridylic acid, trimethylamine, threonine, aspartate, and inosine decreased. Finally, 11 metabolites differed between the HDF and NDF groups, with lactate, butyric acid, succinic acid, and propionic acid upregulated and uric acid, pyruvate, threonine, valine, *β*-glucose, alanine, and aspartic acid downregulated in HDF.

**Table 4:** Comparison of Fecal Metabolite changes in AG and YG Mice

Code	Metabolite	VIP	log_2_(FC)	*P* Value
AG vs YG	Uridylic acid	1.78	1.9561↑	1.28 × 10^8^
	Threonine	1.76	2.8524↑	1.43 × 10^5^
	Propionic acid	1.71	− 1.0176↓	1.09 × 10^6^
	Butyrate	1.67	− 1.4494↓	7.05 × 10^4^
	Inosine	1.64	1.7207↑	2.67 × 10^5^
	Fumarate	1.58	3.0795↑	4.14 × 10^4^
	Leucine	1.55	1.1044↑	5.49 × 10^4^
	pyruvic acid	1.51	3.0353↑	8.65 × 10^5^
	Aspartate	1.47	2.8804↑	1.24 × 10^6^
LDF vs NDF	Uridylic acid	1.8	− 1.5355↓	1.51 × 10^10^
	Glutamate	1.77	2.5244↑	2.63 × 10^10^
	Trimethylamine	1.76	− 2.5236↓	9.96 × 10^9^
	Citric acid	1.66	2.7032↑	9.11 × 10^8^
	Proline	1.65	2.7829↑	3.96 × 10^7^
	Serine	1.65	2.6448↑	8.15 × 10^7^
	Threonine	1.64	− 1.2179↓	1.51 × 10^6^
	Propionic acid	1.63	2.0172↑	2.96 × 10^6^
	Inosine	1.59	− 2.0648↓	1.22 × 10^5^
	Aspartate	1.54	− 1.848↓	3.55 × 10^5^
	Butyrate	1.53	1.5944↑	1.3 × 10^3^
	Fumarate	1.44	− 1.6863↑	3.5 × 10^3^
HDF vs NDF	Uridylic acid	1.72	− 1.4369↓	3.31 × 10^10^
	Threonine	1.69	− 4.834↓	8.11 × 10^9^
	Alanine	1.65	− 1.5114↓	7.13 × 10^7^
	Lactic acid	1.64	1.1799↑	4.19 × 10^6^
	Propionic acid	1.62	1.3613↑	2.03 × 10^5^
	B-glucose	1.58	− 2.837↓	2.67 × 10^5^
	Butyrate	1.54	2.1195↑	1.52 × 10^4^
	Succinic acid	1.52	1.3916↑	7.36 × 10^4^
	pyruvic acid	1.48	− 1.5065↓	1.3 × 10^3^
	Valine	1.45	− 1.1206↓	5.5 × 10^3^
	Aspartate	1.4	− 1.6056↓	0.01

"↑" and "↓" indicate either an increase or decrease of the relative abundance of metabolites in the offspring of DFC intervention

In summary, the OPLS-DA models identified distinct metabolite signatures differentiating the aging, DFC-treated, and young control groups, providing insight into the metabolic pathways altered by aging and DFC supplementation.

### Metabolic pathway analysis of age-related changes and the impact of DFC intake on fecal metabolites

**Fig. 6:**
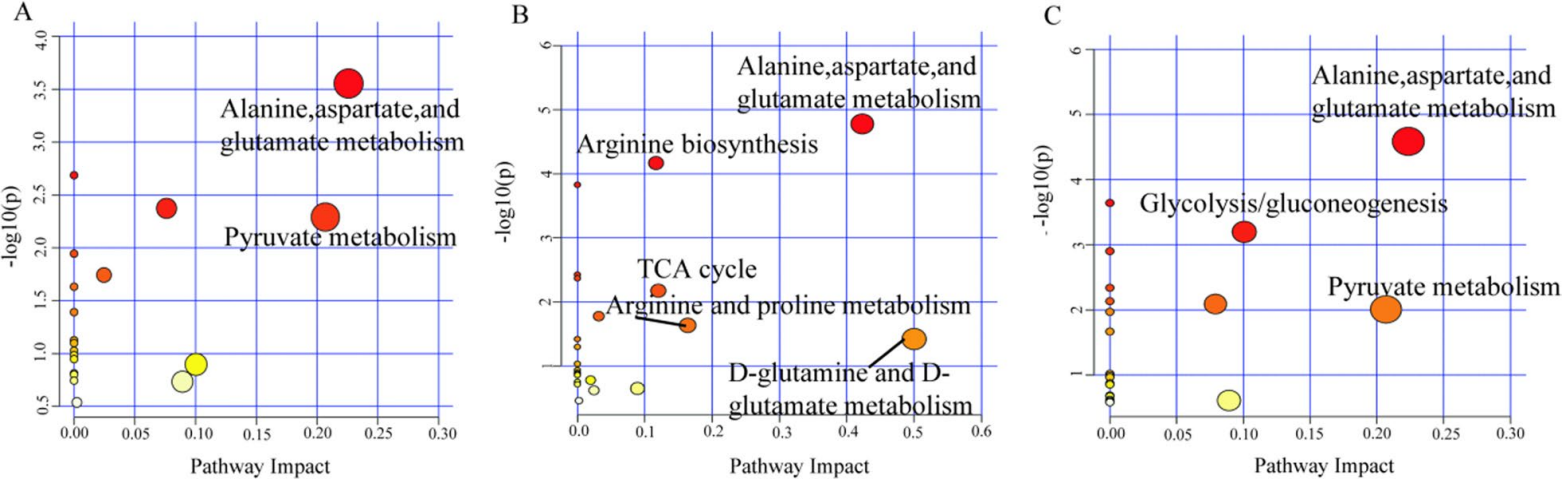
Effect of DFC intake on key metabolic pathways altered in fecal metabolites of mice groups. **A** Metabolic pathways of differential metabolites between AG and YG groups. **B** Metabolic pathways of differential metabolites between LDF and NDF groups (**C**) Metabolic pathways of differential metabolites between HDF and NDF groups. Each bubble represents a distinct metabolic pathway. The Bubble color indicates the *P*-value, and the horizontal axis represents the enrichment factor. *Note* The larger the enrichment factor, the smaller the *P*-value, and the more significant the enrichment degree. Altered metabolic pathways were identified by screening for those with *P* < 0.05 and enrichment factors > 0.1

To gain further insights into the metabolic perturbations underlying the observed metabolite changes with DFC treatment and aging, metabolic pathway analysis was performed on potential differential metabolites (Fig. 6). Between AG and YG, the altered metabolites were enriched in pyruvate metabolism and alanine, aspartate, and glutamate metabolism pathways. Between LDF and NDF, 5 pathways were significantly affected including alanine, aspartic acid and glutamate metabolism, arginine biosynthesis, tricarboxylic acid cycle (TCA), arginine, and proline metabolism, as well as D-glutamine and D-glutamate metabolic pathways. Between HDF and NDF, enriched pathways included pyruvate metabolism, glycolysis/gluconeogenesis, and alanine, aspartate, and glutamate metabolism. The common enriched pathways across the comparisons highlight dysregulation of energy metabolism (TCA cycle, glycolysis/gluconeogenesis) as well as amino acid metabolism mediated by the microbiome (alanine, aspartate, glutamate pathways) with DFC treatment and aging. Specifically, the TCA cycle and glycolysis pathways suggest modulation of carbohydrate metabolism and cellular respiration. Changes in amino acid pathways indicate altered microbial fermentation and inter-conversion of amino acids. Taken together, the metabolic pathway analysis points to significant effects of DFC and aging on gut microbial activity related to carbohydrate and amino acid metabolism.

### Correlation between characteristic bacterial genera and major differential fecal metabolites

**Fig. 7:**
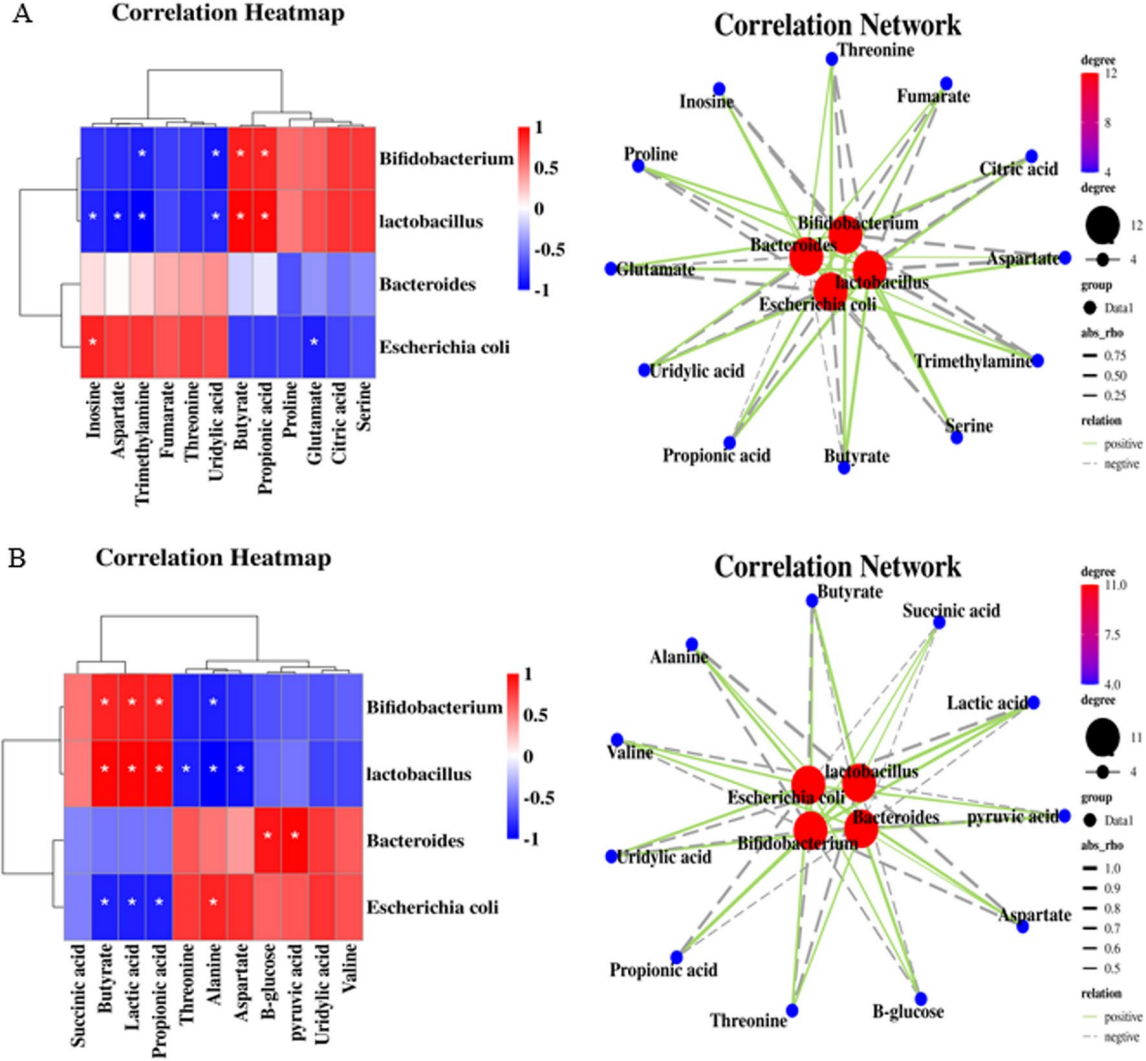
Spearman correlation analysis between characteristic fecal microbiota and main differential metabolites. **A** Heatmap and network diagram showing correlations for the LDF group. **B** Heatmap and correlation network diagram showing correlations for the HDF group. The heatmaps display the Spearman correlation coefficient (R) values, with red indicating a positive correlation and blue indicating a negative correlation. *Note* The network diagrams visualize the correlations, with green lines representing positive correlation, gray lines representing negative correlations, and line thickness proportional to the correlation coefficient. Red circles denote bacteria genera and blue rectangles denote metabolites

To further elucidate the interactions between the characteristic bacterial genera and main differential metabolites, Spearman correlation heatmap and correlation network analysis were performed between the characteristic bacterial genera and the main differential metabolites in each intervention group (Fig. 7).

In the LDF group, *E. coli* was positively correlated with inosine and negatively correlated with glutamate. *Lactobacillus* displayed positive correlations with propionic acid and butyric acid, and negative correlations with aspartic acid, inosine, trimethylamine, and uric acid. *Bifidobacterium* was positively associated with propionic acid and butyric acid, and negatively related to uric acid and trimethylamine.

In the HDF group, *E. coli* showed negative correlations with butyric acid, lactic acid, and propionic acid, and was also negatively correlated with alanine. *Lactobacillus* displayed positive correlations with butyric acid, lactic acid, and propionic acid, while showing negative correlations with threonine, alanine, and aspartic acid. *Bifidobacterium* was positively correlated with butyric acid, lactic acid, and propionic acid, and negatively correlated with alanine. *Bacteroides* exhibited positive correlations with β-glucose and pyruvate.

These association patterns suggest the increased Lactobacillus and *Bifidobacterium* with HDF may drive elevations in short-chain fatty acids through carbohydrate fermentation. The decreased *E. coli* may relieve some of the reductions seen in metabolites like alanine. The higher *Bacteroides* could contribute to increases in lactate, pyruvate, and glucose.

## Discussion

This study utilized a natural aging mouse model to elucidate the regulatory effects of DFC on aging indicators, characteristic bacterial genera, and metabolites. The results demonstrate DFC can delay age-related declines in weight, organ function, and oxidative stress. DFC also promoted the growth of beneficial *Lactobacillus* and *Bifidobacterium* species while inhibiting harmful *E. coli* bacteria. Metabolomics analysis revealed DFC reduces age-associated metabolic dysregulation of carbohydrates, lipids, and amino acids. Correlation analysis explored links between altered metabolites and microbial community changes. Overall, the longevity diet pattern mediated by DFC showed positive regulatory effects on characteristic bacterial genera and metabolites in aging mice, shedding insights into how dietary fiber- characteristic bacterial genera -metabolite interactions impact host health during aging.

Weight is an important indicator of overall health status. Previous studies have shown that mice experience significant memory loss and weight loss decline along with ageing [29]. Yu [24] found that mice fed a standard diet lost significant weight during aging, while mice fed a dietary fiber complex (CDFC) inhibited aging-induced weight loss. In this study, we found the aging control group (AG-NDF) showed a weight loss of 1.60 ± 1.56 g compared to baseline while aging mice fed DFC had reversed the weight loss caused by aging. The high-dose DFC group demonstrated the most substantial effect, indicating DFC alleviated age-related weight loss to maintain health.

Organ coefficient is an important index to evaluate the growth, development, and physiological function of mice. Previous studies have shown that the aging process is characterized by changes in appearance, including a significant decrease in organ coefficients [30]. Here, aging control mice showed reduced organ coefficients, reflecting degeneration. However, 8 weeks of DFC supplementation significantly improved organ coefficients, demonstrating that DFC intake alleviated the rate of organ decline in aging mice.

Antioxidant capacity is an important marker of aging and overall health status. In this study, DFC supplementation did not significantly alter individual antioxidant markers in aging mice. However, overall, there were upward trends in T-AOC, T-SOD, and GSH-Px activities alongside a downward trend in MDA. This suggests DFC may alleviate oxidative stress to some degree, thereby improving overall health levels. DFC's effects on oxidative stress and longevity may be attributable to polyphenols, which are important antioxidants rich in dietary fibers [31, 32]. Our results provide initial evidence that DFC supplementation may mitigate age-related oxidative stress, which is consistent with previous studies. Soluble dietary fiber from passion fruit peel was shown that can prevent oxidative stress induced by DSS in the mouse colon by regulating GSH-Px levels and SOD activity [33]. Similarly, tea residue fiber was reported that increased the levels of SOD and GSH-Px and reduced MDA in diabetes rats [9].

The major impact of DFC supplementation on longevity might be due to a positive regulatory effect on the characteristic bacterial genera. Previous studies have shown dietary fibers can selectively enrich beneficial bacteria. For example, a dietary fiber composite (oat corn konjac, oat bran, skimmed milk, whole kudzu root, pumpkin) selectively enriched beneficial bacteria such as *Bifidobacterium*, *Akkermansia*, and *Rombutzia* in obese mice [34]. Chitosan promoted the growth of the *Lactobacillus* genus while inhibiting the *Enterobacterium* and *Enterococcus* [35]. Stachyose α-Oligosaccharides also increased beneficial *Lactobacillus* and *Bifidobacterium* and inhibited the growth of pathogenic *Enterobacteriaceae* [36]. Similarly, our study showed that 8 weeks of DFC intake increased beneficial *Lactobacillus* and *Bifidobacterium* genera in aging mice by 329.73% and 239.75% respectively, while decreasing the harmful bacteria *E. coli* by 45.62%, *Lactobacillus* and *Bifidobacterium* are considered the most typical probiotics [37], producing short chain fatty acids such as acetic acid, propionic acid, and butyric acid to promote intestinal health [38, 39]. In summary, DFC supplementation significantly promoted the growth of advantageous bacteria (*Lactobacillus* and *Bifidobacterium*) in the intestine of aging mice, while suppressing undesirable bacteria (*E. coli*) and *Bacteroides*.

The mechanisms by which the DFC intake improved intestinal health and longevity are closely correlated to alterations of metabolic pathways. Compared to young mice, aging mice showed upregulation of seven differential metabolites including uric acid, aspartic acid, threonine, inosine, pyruvate, and fumarate, while downregulation of propionic acid and butyric acid. They are metabolites relating to glycolysis downstream of pyruvate, players involved in the TCA cycle, and lipid and amino acid metabolism pathways. These changes indicated that aging mice may experience dysregulation of energy production, lipid metabolism disorders, and inflammatory reactions. Our study indicated that DFC supplementation could effectively reverse most of the above metabolic changes, demonstrating significant effects on microbial carbohydrate and amino acid metabolism related to aging and gut health.

**Fig. 8:**
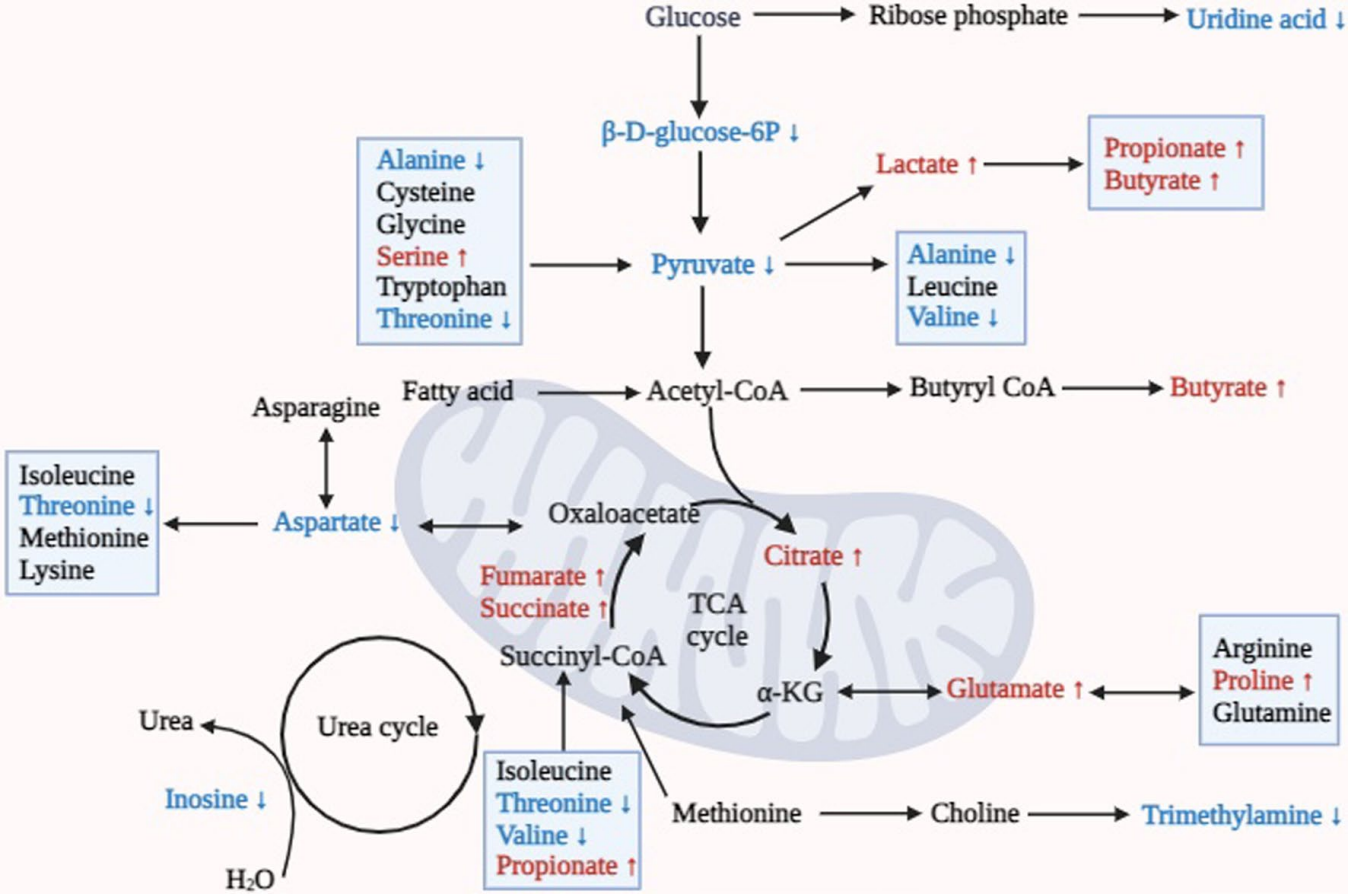
Summary of metabolic pathways of the DFC involved in glucose lipid metabolism and amino acid metabolism. Pyruvate and glycolysis/glycolysis pathways are the main pathways regulating glucose metabolism and energy metabolism, and the lactate produced by this pathway can be converted to other short-chain fatty acids such as propionic acid and butyric acid. The alanine, aspartate, and glutamate pathways produce pyruvate and gibberellic acid, respectively, which participate in the citric acid cycle for energy production, with gibberellic acid, citric acid, and succinic acid being intermediates of the TCA cycle. *Note* Red = upregulated metabolites; Blue = downregulated metabolites

DFC-induced metabolic changes appear to counteract some of the age-associated disruptions in microbial community function. For example, the upregulation of uric acid and increased fecal short-chain fatty acids like propionic acid and butyric acid are known to be related to the growth of certain intestinal bacteria, which are consistent with previous studies [40, 41]. Pathway analysis further supports DFC modulation of key metabolic processes. As summarized in Fig. 8, alanine, aspartic acid, and glutamate generate pyruvate and fumarate, participating in the TCA cycle to generate energy. Dysregulated glycolysis and glutathione metabolism can lead to lipid accumulation and oxidative stress that contribute to aging [42–45]. After DFC intervention, the levels of trimethylamine, valine, threonine, and inosine were down regulated, while the levels of fumarate and citric acid were upregulated. Decreased threonine and inosine levels suggest DFC may reduce risks of inflammatory conditions like irritable bowel syndrome (IBD) and ankylosing spondylitis (AS) [26, 27]. The reduction in valine levels means that DFC may be associated with a reduced risk of diabetes [46]. As trimethylamine is considered a risk marker for cardiovascular disease [47], DFC intervention appears to improve cardiovascular health as well.

In summary, DFC-induced alterations in TCA cycle intermediates, lipid metabolism, and amino acid metabolism likely counteract age-related metabolic dysfunction, which is mediated through modulation of the microbiome. The complex in vivo metabolic impacts of DFC underscore the intricate interplay between diet, microbiome, and host.

## Conclusion

Our multi-dimensional analysis shows DFC supplementation can favorably reshape microbial-host metabolic interactions during aging through specific pathways. The longevity diet pattern established here supplies a framework for optimizing dietary fiber content and composition to benefit the characteristic bacterial genera and host metabolism throughout life. Further research is warranted to build upon these findings and translate them into dietary recommendations and interventions that promote healthy aging in humans. However, this study only investigated the changes in four characteristic bacterial genera. Future research can determine the overall regulation of gut microbiota through high-throughput sequencing technology to further reveal the interaction between dietary fiber, gut microbiota, and host.

### Supplementary Information

**Additional file 1. Table S1.** English comparison table of abbreviations of paper terms.

**Additional file 2. Table S1.** The feed formulation for each group of mice used in this study.

**Additional file 3. Table S1.** ^1^H-NMR signal assignment of main metabolites in mouse feces. **Fig. S1.** OPLS-DA scores plots of fecal metabolites between different mouse groups and cross validation by Permutations tests. (A) OPLS-DA plot of AG versus YG (R^2^Y = 0.994, Q^2^ = 0.991) shows distinct clustering and separation of fecal metabolites by age. (B) OPLS-DA plot of LDF and NDF (R^2^Y = 0.994, Q^2^ = 0.987) shows separation by diet. (C) OPLS-DA plot of HDF and NDF (R^2^Y = 0.994, Q^2^ = 0.987) shows more distinct separation by diet. R^2^Y and Q^2^ values > 0.5 indicate a high degree of model fit. Permutations testing shows all blue simulated Q^2^ values (left) are lower than the original point (right) and the regression line intercept is < 0.5, indicating the OPLS-DA models effectively discriminate between groups.

## Data Availability

The data in this study are available on request from the author.
