# Supplementary material for: Exploring the effects of the dietary fiber compound mediated by a longevity dietary pattern on antioxidation, characteristic bacterial genera, and metabolites based on fecal metabolomics
Source: Nutr Metab (Lond). 2024 Apr 4;21:18. doi: 10.1186/s12986-024-00787-y (PMC10993571; doi:10.1186/s12986-024-00787-y)
Supplement: Supplementary file 1 — Additional file 1. Table S1. English comparison table of abbreviations of paper terms. [file 12986_2024_787_MOESM1_ESM.docx]

| **#** | **Full name** | **Abbreviation** |
| --- | --- | --- |
| 1 | dietary fiber compound | DFC |
| 2 | no dietary fiber subgroup | NDF |
| 3 | low DFC dose subgroup | LDF |
| 4 | high DFC dose subgroup | HDF |
| 5 | aged group | AG |
| 6 | young control group | YG |
| 7 | aging mice group on a no dietary fiber feed | AN |
| 8 | aging mice group on a low-dose DFC feed | AL |
| 9 | aging mice group on a high-dose DFC feed | AH |
| 10 | young mice group on a no dietary fiber feed | YN |
| 11 | insoluble dietary fiber | IDF |
| 12 | soluble dietary fiber | SDF |
| 13 | total antioxidant capacity | T-AOC |
| 14 | total superoxide dismutase | MDA |
| 15 | glutathione peroxidase | GSH-Px |
| 16 | total superoxide dismutase | T-SOD |
| 17 | quantitative real-time PCR | qPCR |
| 18 | nuclear magnetic resonance hydrogen spectroscopy | 1H NMR |
| 19 | escherichia coli | *E. coli* |
| 20 | diamine oxidase | DAO |
| 21 | trimethylamine nitrogen oxides | TMAO |
| 22 | principal component analysis | PCA |
| 23 | orthogonal partial least squares discriminant analysis | OPLS-DA |
| 24 | kyoto encyclopedia of genes and genomes | KEGG |
| 25 | human metabolome database | HMDB |
| 26 | Biological Magnetic Resonance Bank | BMRB |
| 27 | log2Fold Change | log2FC |
| 28 | variable importance in projection | VIP |
| 29 | tricarboxylic acid cycle | TCA |
| 30 | short chain fatty acids | SCFA |

**Table S1** English comparison table of abbreviations of paper terms
