# Supplementary material for: Exploring the effects of the dietary fiber compound mediated by a longevity dietary pattern on antioxidation, characteristic bacterial genera, and metabolites based on fecal metabolomics
Source: Nutr Metab (Lond). 2024 Apr 4;21:18. doi: 10.1186/s12986-024-00787-y (PMC10993571; doi:10.1186/s12986-024-00787-y)
Supplement: Supplementary file 2 — Additional file 2. Table S1. The feed formulation for each group of mice used in this study. [file 12986_2024_787_MOESM2_ESM.docx]

| **Group** | **Ingredient** | **gm%** | **kcal%** |
| --- | --- | --- | --- |
|  | Casein, 30Mesh | 140 | 560 |
|  | L-Cystine | 1.8 | 7.2 |
|  | Corn Starch | 495.692 | 1983 |
|  | Maltodextrin 10 | 125 | 500 |
|  | Sucrose | 100 | 400 |
| NDF | Cellulose | 50 | 0 |
|  | Soybean Oil | 40 | 360 |
|  | t-Butylhydroquinone | 0.008 | 0 |
|  | Mineral Mix S10022M | 35 | 0 |
|  | Vitamin Mix V10037 | 10 | 40 |
|  | Choline Bitartrate | 2.5 | 0 |
|  | Total | 1000 | 3850 |
|  | Casein, 30Mesh | 126 | 504 |
|  | L-Cystine | 1.62 | 6.48 |
|  | Corn Starch | 446.1228 | 1784.7 |
|  | Maltodextrin 10 | 112.5 | 450 |
|  | Sucrose | 90 | 360 |
|  | Cellulose | 45 | 0 |
| LDF | Soybean Oil | 36 | 324 |
|  | t-Butylhydroquinone | 0.0072 | 0 |
|  | Mineral Mix S10022M | 31.5 | 0 |
|  | Vitamin Mix V10037 | 9 | 36 |
|  | Choline Bitartrate | 2.25 | 0 |
|  | 10%DFC | 100 | 246.35 |
|  | Total | 1000 | 3711.53 |
|  | Casein, 30Mesh | 112 | 448 |
|  | L-Cystine | 1.44 | 5.76 |
|  | Corn Starch | 396.5536 | 1586.4 |
|  | Maltodextrin 10 | 100 | 400 |
|  | Sucrose | 80 | 320 |
|  | Cellulose | 40 | 0 |
| HDF | Soybean Oil | 32 | 288 |
|  | t-Butylhydroquinone | 0.0064 | 0 |
|  | Mineral Mix S10022M | 28 | 0 |
|  | Vitamin Mix V10037 | 8 | 32 |
|  | Choline Bitartrate | 2 | 0 |
|  | 20%DFC | 200 | 492.70 |
|  | Total | 1000 | 3572.86 |

**Table S2** The feed formulation for each group of mice used in this study
