# Supplementary material for: Exploring the effects of the dietary fiber compound mediated by a longevity dietary pattern on antioxidation, characteristic bacterial genera, and metabolites based on fecal metabolomics
Source: Nutr Metab (Lond). 2024 Apr 4;21:18. doi: 10.1186/s12986-024-00787-y (PMC10993571; doi:10.1186/s12986-024-00787-y)
Supplement: Supplementary file 3 — Additional file 3. Table S1. 1H-NMR signal assignment of main metabolites in mouse feces. Fig. S1. OPLS-DA scores plots of fecal metabolites between different mouse groups and cross validation by Permutations tests. (A) OPLS-DA plot of AG versus YG (R2Y = 0.994, Q2 = 0.991) shows distinct clustering and separation of fecal metabolites by age. (B) OPLS-DA plot of LDF and NDF (R2Y = 0.994, Q2 = 0.987) shows separation by diet. (C) OPLS-DA plot of HDF and NDF (R2Y = 0.994, Q2 = 0.987) shows more distinct separation by diet. R2Y and Q2 values > 0.5 indicate a high degree of model fit. Permutations testing shows all blue simulated Q2 values (left) are lower than the original point (right) and the regression line intercept is < 0.5, indicating the OPLS-DA models effectively discriminate between groups. [file 12986_2024_787_MOESM3_ESM.docx]

**Table S1** ^1^H-NMR signal assignment of main metabolites in mouse feces

| **#** | **metabolite** | **chemical shiftδ^1^H** | **#** | **metabolite** | **chemical shiftδ^1^H** |
| --- | --- | --- | --- | --- | --- |
| 1 | Bile acid | 0.69(s) | 24 | Asparagine | 3.04(m) |
| 2 | Butyric acid | 0.9(t) | 25 | Histidine | 3.15(m) |
| 3 | Isovaleric acid | 0.91(d) | 26 | Choline | 3.21(s) |
| 4 | Isoleucine | 0.93(t),1.01(d) | 27 | Methanol | 3.37(s) |
| 5 | α-ketoisocaproate | 0.94(d),1.13(d),2.63(d), 3.03(m) | 28 | Glycine | 3.58(s) |
| 6 | Leucine | 0.96(t) | 29 | Threonine | 3.60(d)、4.24(m) |
| 7 | Valine | 0.99(d),1.04(d),2.27(m), 3.62(d) | 30 | β-glucose | 3.25(dd),3.50(t),3.75(m), 3.91(dd),4.65(d) |
| 8 | Propionic acid | 1.06(t),2.19(q) | 31 | α-Glucose | 3.42(dd),3.55(m),3.84(dd),5.24(d) |
| 9 | Lactic acid | 1.34(d),4.12(q) | 32 | a-xylose | 3.52(d) |
| 10 | Alanine | 1.49(d),3.78(m) | 33 | Glutamate | 3.76(m) |
| 11 | Citrulline | 1.56(m) | 34 | α-galactose | 3.81(dd) |
| 12 | Lysine | 1.72(m),1.90(m) | 35 | β-galactose | 3.65(dd),4.59(d),3.94(dd) |
| 13 | Acetic acid | 1.92(s) | 36 | Serine | 3.99(m) |
| 14 | N-Acetyl Glycoprotein | 2.06(s) | 37 | 1,3-Dihydroxyacetone | 4.43(s) |
| 15 | Methionine | 2.15(s) | 38 | Glycogen | 5.42(m) |
| 16 | Proline | 2.36(m),3.34(m) | 39 | Inosine | 6.12(d) |
| 17 | Pyruvate | 2.39(s) | 40 | Fumaric acid | 6.52(s) |
| 18 | Succinic acid | 2.42(s) | 41 | Tyrosine | 6.90(d),7.20(d) |
| 19 | Glutamine | 2.45(m) | 42 | Uridine | 7.35(d),7.42(s) |
| 20 | Citric acid | 2.65(d) | 43 | Uracil | 5.81(d),7.54(d) |
| 21 | Dimethylamine | 2.70(s) | 44 | Xanthine | 7.89(s) |
| 22 | Aspartate | 2.68(m),2.82(dd),3.91(dd) | 45 | Hypoxanthine | 8.19(s),8.21(s) |
| 23 | Trimethylamine | 2.88(s) | 46 | Formic acid | 8.46s |


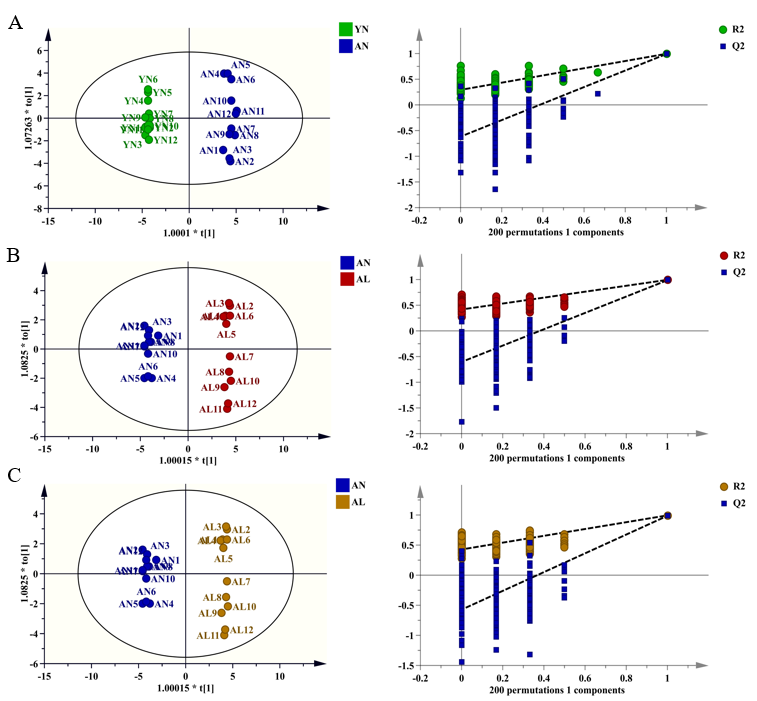


**Fig. S1** OPLS-DA scores plots of fecal metabolites between different mouse groups and cross validation by Permutations tests. (A) OPLS-DA plot of AG versus YG (R^2^Y=0.994, Q^2^=0.991) shows distinct clustering and separation of fecal metabolites by age. (B) OPLS-DA plot of LDF and NDF (R^2^Y=0.994, Q^2^=0.987) shows separation by diet. (C) OPLS-DA plot of HDF and NDF (R^2^Y=0.994, Q^2^=0.987) shows more distinct separation by diet. R^2^Y and Q^2^ values > 0.5 indicate a high degree of model fit. Permutations testing shows all blue simulated Q^2^ values (left) are lower than the original point (right) and the regression line intercept is < 0.5, indicating the OPLS-DA models effectively discriminate between groups.
